# Primate Sex and Its Role in Pleasure, Dominance and Communication

**DOI:** 10.3390/ani12233301

**Published:** 2022-11-26

**Authors:** Esther Clarke, Katie Bradshaw, Kieran Drissell, Parag Kadam, Nikki Rutter, Stefano Vaglio

**Affiliations:** 1 Department of Linguistics and Philosophy, Massachusetts Institute of Technology, Cambridge, MA 02139, USA; 2 Department of Anthropology, Durham University, Durham DH1 3LE, UK; 3 Warnell School of Forestry and Natural Resources, University of Georgia, Athens, GA 30602, USA; 4 Department of Sociology, Durham University, Durham DH1 3HN, UK

**Keywords:** non-human primates, sexual behaviour, sexual experience, sexual intercourse, sexual pleasure

## Abstract

**Simple Summary:**

Sex is inextricably linked to reproduction, but in humans at least it also has many other roles. In the animal kingdom sex has traditionally been considered as having only a reproductive function. However, many animal species regularly engage in sex outside of their fertile period, and homosexual and immature sex are more common than previously thought. This suggests that sex has also an important role outside of reproduction. Is the human sexual experience, which includes pleasure, dominance, and communication, unique, or do other animals also share these experiences to any extent? If so, is there any way to measure them, or are they beyond the rigour of scientific objectivity? What would be the evolutionary implications if human-like sexual experiences were found amongst other animals? In this commentary we attempt to address these questions looking at the evidence from our relatives, non-human primates, and suggest potential methods for measuring some of these experiences empirically.

**Abstract:**

Sexual intercourse in the animal kingdom functions to enable reproduction. However, we now know that several species of non-human primates regularly engage in sex outside of the times when conception is possible. In addition, homosexual and immature sex are not as uncommon as were once believed. This suggests that sex also has important functions outside of reproduction, yet these are rarely discussed in sex-related teaching and research activities concerning primate behaviour. Is the human sexual experience, which includes pleasure, dominance, and communication (among others) unique, or do other primates also share these experiences to any extent? If so, is there any way to measure them, or are they beyond the rigour of scientific objectivity? What would be the evolutionary implications if human-like sexual experiences were found amongst other animals too? We comment on the evidence provided by our close relatives, non-human primates, discuss the affective and social functions of sex, and suggest potential methods for measuring some of these experiences empirically. We hope that this piece may foster the discussion among academics and change the way we think about, teach and research primate sex.

## 1. Introduction

Sex as a form of reproductive activity is a complex concept, and a relative conundrum [1,2]. A recent scientific foray into the diverse nature of sex brings up an inescapable contradiction – that sexual reproduction actually reduces numbers by fusing two gametes into one [3]. In addition, sex typically involves a random process of recombination of DNA or RNA sequences. This process may create novel and beneficial allele combinations, but it can also break up existing favourable combinations [4]. The classic theory of sexual selection is based on the discrepancy that male and female animals face in the cost of reproduction: males should mate with as many females as possible in order to maximize reproductive success, whilst females should ideally mate with a male with “good genes” to maximize offspring survival [5]. This differential cost is also faced by male and female humans [6]. With its random nature, costs and potential for maladaptation, some scholars have wondered why sex exists at all, especially when there are far more predictable forms of asexual reproduction that avoid the “two-fold cost of sex” [7]. One well-known explanation is that sex introduces novel allele combinations that keep organisms one step ahead of parasites – dubbed the Red Queen Hypothesis which has gleaned empirical support [1,8]. Whatever the benefits/drawbacks of sexual over asexual reproduction, there is a huge field of research surrounding behavioural patterns associated with sex. The division of the sexes has led to the evolution of a host of mating strategies, presumably designed to ensure the replication of genes in a changing environment [1]. In order for sexual reproduction to be maintained, its benefits must outweigh its costs; however, these are not always simple and self-evident, particularly when dealing with highly intelligent and autonomous animals.

The focus of this paper is on primates, including humans, who familiarly engage in sexual reproduction with two sexes. The promise of randomly recombined alleles (resulting in genetically diverse offspring) is argued to be the driving force behind primates’ engagement in sexual behaviours, and harks back to Darwin’s early theories on sexual selection [9]. The bedrock of modern primate behavioural ecology includes the socioecological model [10,11], tying the majority of primate behaviours, social systems, and physiology to ultimate survivalist and reproductive functions [9]. While these offer likely explanations for many traits (i.e., mate choice, mating system, sexual dimorphism, infanticide, etc.), there are always exceptions that warrant further investigation. One such exception is engaging in sex when the chances of producing offspring are low or zero. This was noted over a century ago in mammals and referred to as “abnormal oestrus” by Heape [12], who concluded that “the period of oestrus is not invariably identical with the period of ovulation; the two are separate functions, possibly closely associated, but also possibly widely divergent” (pp 33). This uncoupling of ovulation and sexual behaviour was also noted by Hrdy [13]: “In the course of primate evolution, there has apparently been a trend away from strictly hormonal determination of receptivity” (pp 33). Indeed, bonobos’ sexual swellings are only loosely correlated with ovulation [14]. Moreover, olive baboons engage in copulation when sexual swellings are at their flattest and females are non-fertile [15], again suggesting sexual attraction is not strictly determined by fertility, and in this case, not even determined by the presence of tumescent sexual swellings. Wallen [16], who states that in primates “copulatory abilities have become emancipated from hormonal control” (pp 339), suggests that social context/complexity determines a primate’s sexual schedule; sex only follows the female’s ovulatory cycle when social pressures exist to prevent otherwise free mating.

We do not assume that non-human primates are consciously aware of the possibility that sexual behaviour can lead to offspring (in fact, some human societies seem to have no awareness of this fact [17]). We also acknowledge that optimal timing for sex (i.e., when reproduction is most likely) influences a host of physiological co-occurrences that do make sex more attractive. Some examples include hormone-mediated sexual swellings across a range of primate species [18], courtship displays/approaches in chimpanzees [19,20], odour signals in lemurs, marmosets, tamarins, macaques and baboons [15,21,22,23] etc. Yet, as we said above, sex occurs outside of the optimal time periods for reproduction, even when there is little or no chance of conception. Nonetheless, since an ultimate function of sex is reproduction, scholars, and higher education texts, are apt to couch analyses of all primate sexual behaviours in terms that appear to maximize reproductive potential, including offspring survival i.e., the ultimate evolutionary perspective [24]. For example, scholars cite the sexual selection hypothesis to explain female promiscuity, and sperm competition to explain mate guarding and male promiscuity (reviewed in [25]).

In this paper we review the non-reproductive functions of sexual behaviour in primates, couching them instead in affective terms, such as pleasure, dominance, and communication. While traditionally allocating affect to non-human animals has been hotly debated, it is becoming more acceptable [26]. This approach may prove useful in describing behaviours including sex where, for example, an “impending ovulation” cue is not enough to explain a repeated and sought-after action. The evolutionary perspective is not incompatible with affective terminology or theory. Instead, we explore the possibility that these affective explanations are by-products or secondary adaptations to the ultimate function of sex, as some argue is the case with the diverse nature of contemporary human sexual behaviour [17,27].

Specifically, this commentary explores the following:

- Non-conceptive sex
- Sex for pleasure
- Sex as a means to dominance and protection from infanticide
- Sex as a means of communication (reconciliation)
- Other influencing factors

## 2. Non-Conceptive

Evidence for non-conceptive sexual activity is widespread in the animal kingdom [28,29]. In particular, sex during pregnancy, same-sex encounters, oral sex, and masturbation are observed in almost all non-human animals, including primates [28,30]. There are several hypotheses to explain these encounters, which do not appear to have an ultimate evolutionary mechanism. These include the “practice-for-heterosexual-sex” hypothesis [31], the “developmental-by-product” hypothesis [32,33,34], and the suggestion that these sexual behaviours are primarily “motivated” by social interactions, such as between dominant and subordinate individuals, as well as between individuals who are forming coalitions [35]. However, studies on male-male sexual behaviours in Japanese macaques [29] suggest that these can be considered homosexual behaviours rather than socio-sexual behaviours (i.e., sexual in terms of their superficial form, but enacted to facilitate adaptive social goals) [18]. Additionally, recent findings on female-male, female-female, and male-female mounting displayed as cultural sexual practices and supernormal courtship behavioural patterns in Japanese macaques [36,37], and on stone tool-assisted masturbation in long-tailed macaques [38]), emphasize the sexual nature of non-conceptive behaviours, rather than socio-sexual behaviours, in Old World primates. 

These non-conceptive behaviours suggest that both male and female animals benefit from some physiological and/or neurological reward from sex, likely mediated via the dopaminergic pathway.

## 3. Sex for Pleasure

Lack of available data is the main problem when including pleasure in models of sexual behaviours, though sensory pleasure is not outside the realms of scientific inquiry [39]. Nevertheless, there has been a focus on ultimate evolutionary (procreative) contexts over proximate, experiential ones [40]. The presence of non-conceptive sex can be considered an adaptation to social settings [16], and to facilitate pair bonds [23,41], but the mechanisms of pleasure might account for certain drives in behavioural differences. Knowledge of reproductive physiology of species and specific life histories of study populations [42], along with data on change in neuromodulator hormones like oxytocin, vasopressin, and oestrogen, which are related to experiential pleasure [43], are necessary for a meaningful inclusion of pleasure in research. The current lack of evidence in the latter can be improved through invasive (blood), non-invasive (urine, faeces, saliva, hair; [42]) or minimally invasive (wireless telemetry [44]) physiological monitoring methodologies. 

The difficulty with non-invasive sampling for detecting oxytocin in urine samples is that high accuracy in preparing the hormonal assays is needed, due to very low amounts of excreted oxytocin [42]. Despite these challenges, minimally invasive measures are favourable and, in fact, critical given the great majority of primate species being classified as vulnerable to critically endangered. Furthermore, serum measures of hormones may be less accurate than urinary measures, since many hormones are released as pulses into the bloodstream, whereas the bladder serves to integrate hormonal release over time. Nevertheless, the use of continuous remote hormonal monitoring (biotelemetry) is a middle ground between invasive and non-invasive technologies that may prove very fruitful [45]. However, one way of including pleasure in investigations of sexual behaviours without including an “ultimate reproductive function” is using these methodologies for researching non-conceptive sexual behaviours like non-copulatory mountings, oral sex, genital and anal stimulation using fingers, auto-erotic behaviours, same-sex interactions, group sex, and contextual orgasms in laboratory and wild settings [39]. For instance, same-sex mounting interactions in animals have traditionally been characterized as socio-sexual; however, Vasey’s research provides direct evidence concerning the sexual nature of female-female mounting in Japanese macaques and supports the conclusion that these interactions can be considered homosexual behaviour with a hedonic component [46,47,48,49]. 

The nerve structures within both the male and female sexual organs are highly similar, at least in humans [27], suggesting that sex is appetitive for both sexes. It seems intuitive that primates experience sexual pleasure in a similar way to humans [50,51]. Indeed, female orgasms occur in lemurs, marmosets, macaques, and apes [18,28]. One explanation for female orgasm is the multiple partner hypothesis [52] according to which, female orgasms are achieved through copulation with multiple partners; as the female is constantly being stimulated through repeated sexual intercourse, she eventually achieves an orgasm. However, it is not clear how these theories could explain orgasm in primate species that are socially monogamous and where neither the male nor female is dominant (e.g., titi monkeys and gibbons). In the case of gibbons, long-term associations (pair bonds) may be strengthened by non-conceptive sex [53] and are likely facilitated by sexual behaviour [41], while extra-pair copulations with other males of equal rank may also be explained by infanticide avoidance, according to the sexual selection hypothesis [54]. 

If primates had sex mainly for pleasure, what would be the mechanisms that result in females of some species appearing to selectively mate with higher ranking males [55,56], males with visual cues such as brighter faces (e.g., mandrills: [57]), and even males who are currently subordinate but seem likely to ascend to dominance relatively soon [58]? It seems likely that sexual pleasure is a by-product that makes sex attractive at times when reproduction is not an option.

Georgiadis and colleagues [27] suggests that sexual behaviours follow a “pleasure cycle” as with food: “wanting”, “liking” and “inhibition”. Dopamine is present in the “wanting” stage of motivated behaviour, whereas prolactin and potentially the “feel good” hormone, oxytocin, could be part of the “liking” stage. For example, both prolactin and oxytocin are released during orgasm in men and women (reviewed in [59,60]), while in tamarins, pairs with higher sexual activity have higher levels of prolactin and oxytocin [61], suggesting these hormones are involved in sexual reward mechanisms. In addition, marmosets have been experimentally conditioned to expect sex in association with novel olfactory stimuli, such that the odour alone can elicit male erections, even without the presence of a partner [62]; a conditioned sexual response based on sexual reward is likely a clear indication of pleasure. To empirically determine whether female (or male) primates derive pleasure from sex, we need stringent methods of assessment. Currently available measures could determine the physiological effects of sexual stimulation (as previously mentioned), but we need to also examine their relationship with the above socio-sexual contexts, for example, the link between male dominance and female pleasure.

## 4. Sex as a Means to Dominance and Protection from Infanticide

Dominance is inherently involved in sex within primate societies and social structure. For example, the high-ranking male often has almost unrivalled access to the females of his choice. In some primate species, such as capuchin monkeys, alpha males sire many more offspring than predicted by chance [63] and females actively elicit sex from the alpha male [64]. Additionally, low-ranking female Japanese macaques prefer to copulate with the dominant male rather than low-ranking males, to obtain both direct (i.e., services) and indirect (i.e., “good genes”) benefits, but also because male rank is correlated with the amount of orgasms a female achieves [65].

However, sex also has a different, less pleasurable, function with regard to dominance. Sexual coercion (for instance, behaviours such as mate-guarding, physical prevention of other male access to a female, aggressively punishing promiscuity, etc.) and infanticide are present amongst primate societies, including humans. For example, female orangutans are sometimes forced to have sex with sub-adult/unflanged males [66]. While the function of sexual coercion may be to increase the probability of siring offspring [67], it may also be to exert dominance in its absence (e.g., unflanged orangutan males are often low-ranking and do not normally have as much access to females as flanged males). 

Infanticide is a by-product of reproductive sex. While itself is not a function of sex, sexual strategies (e.g., promiscuity and resultant paternity confusion) taken by females to mitigate its risk undoubtedly are [13]. The female receives benefits from confusing the paternity of the males (i.e., to prevent infanticide) [68], as well as gaining material benefits from having orgasms when the male partner ejaculates and, in addition, having multiple partners (i.e., to protect from other males) [5,69]. While investigating all the possible functions of infanticide are outside the scope of this paper, we feel it prudent to mention that female promiscuity, as a form of paternity confusion, is reliant on males being unable to recognize their own offspring, using mating history to assess paternity instead [56]. Some evidence for this comes from baboons [70]. However, there is also evidence to suggest that some male primates can recognize their offspring, even amongst societies where infanticide risk is relatively high (e.g., chimpanzees: [71]). In other primate species there is also evidence of kin recognition (e.g., macaques: [72,73], mandrills, hanuman langurs, baboons and capuchins: reviewed in [74]). Therefore, paternity confusion may not always be a reliable end-product of female promiscuity. 

Male-female associations, or “friendships”, are present among several primate species [70] and include: olive baboons [75,76]; chacma baboons [77]; yellow baboons [78,79]; barbary macaques [80,81]; and Rhesus macaques [82]. These friendships are thought to protect infants from infanticide, and are thus highly adaptive for the female [77]. Recent evidence suggests that these associations can be stable and favour a “mate-then-care” hypothesis for males [70]. In gregarious primates, friendships can form between multiple partners and in these cases, sex can be used as a communicative tool to both form and reconcile friendships.

## 5. Sex as a Means of Communication (Reconciliation)

In humans “makeup sex” is a term often used in modern culture to describe the act of sexual intercourse as a way to minimize tension caused by agonistic situations, normally following an argument with a partner [83]. In this sense, sex acts as a communication strategy, a way of calming a situation down without the need of words or a form of reconciliation (ibid). This occurs also in non-human primates. Most reported examples of sex for communication come from bonobos. Wrangham (1993) defines three ways in which sex amongst bonobos can be described as a form of communication: (i) to form friendships and relationships; (ii) when expecting agonistic interactions; and (iii) to reconcile following agonistic interactions – with at least two ways [(i) & (iii)] requiring pleasure as a crucial component to make possible the communication itself [84]. de Waal and Lanting report that sexual behaviour in bonobos occurs frequently as a form of relieving social tensions, amongst males-females, males-males, and females-females in both adults and juveniles [85].

However, as mentioned previously, non-conceptive sexual behaviour also occurs in other primate species. There is some evidence that sex outside of fertile periods can restore relationships and reduce stress; for example, increased sexual activity follows short separations [86], or the odour of a novel ovulating female [23] in socially biparental and cooperatively breeding primates, such as marmosets. Regular male-female relations outside of fertile periods are reported in white-faced capuchins with, in addition, female-female and male-male sexual contacts [87]. The authors hypothesize that male-male sexual behaviour in white-faced capuchins could have acted as a form of communication because the contact often took place following tension within the group, like bonobos. Tibetan macaques have also been found to have non-conceptive sexual relations, with male-female sexual behaviours being observed outside of the fertility period [88]. These occurrences also took place following disagreements within the groups, as well as prior to food sharing; however, in this case the authors do not interpret it as a form of communication. Stump-tailed macaques perform highly conspicuous socio-sexual behaviours during post-conflict reunions [89]. The authors highlight the meaning of communication with a focus on reconciliation via multiple post-conflict triadic affiliations. In addition, Bailey and Zuk review and highlight instances of same-sex sexual behaviour across a wide range of species outside of primates, including bottlenose dolphins, chinstrap penguins and Laysan albatross [30]. The authors cite several reasons as to why this may occur, from forming relationships, practicing for later reproductive events and to reduce tensions – but again do not refer to communication. It is therefore interesting to highlight that the most widely cited form of sex as a means of communication, outside of humans (bonobos), appears to be extrinsically linked to the reduction of tension as a tool for forming relationships. However, in other instances, where sex appears to serve the same purpose in other non-human animal species, it is not widely cited as a communication strategy. As a result of this, there appears to be a lack of research that relates or is willing to relate sexual behaviour to communication across species. In addition, there is an over-interpretation in chimpanzees and humans, in stark contrast to the intrinsic role sex appears to play in communication strategies of bonobos. 

Thus, future research within this area needs to explore the role that sex plays within the social systems of species other than humans and bonobos with an experimental focus on its communicative function. For instance, if sex is being cited as an action to reduce tension within groups, what are the outcomes of this? Do the outcomes correlate with the results of other forms of communication during agonistic interactions and, if so, is sex in this context also communicative? Alternatively, where sex is used as a trade-off for food sharing in primate species [90,91,92], how does this differ from forms of gestural communication and grooming as means of acquiring objects? Such future research is needed to examine whether sex plays a role in communication across the primate taxa and the entire animal kingdom. Aside from these important communicative functions, there are also other additional factors that may influence non-conceptive sex in primates.

## 6. Other Influencing Factors

Female primates that conceal their ovulation have been hypothesised to do so to confuse paternity [52]. However, evidence suggests that concealed ovulation may instead promote paternal care and confidence [93]. This is another interesting potential explanation for non-conceptive sex – since males do not know when conception is possible, females are able to ensure male commitment when biparental offspring care is necessary, and therefore concealed ovulation may have led to monogamy.

Furthermore, the type of mating and social system may influence the frequency and function of non-conceptive sex [94]. For instance, primate groups that favour cooperative breeding or monogamy may be more likely to show evidence of communicative/reconciliatory sex (since there may be more incentives to invest in long-term relationships), whereas in polygynous primates, non-conceptive sex may be more likely to be related to dominance and infanticide avoidance (since relationships may be transient and/or rank-dependent). Similarly, whether societies are patriarchal or matriarchal may impact sexual behaviours and the roles of these behaviours. In this case, the costs of sexual behaviours and resultant benefits may vary wildly dependent on the societal structure, though not enough is known to make accurate predictions.

It is worth noting that while other forms of non-conceptive sex have the potential to become conceptive (i.e., sex outside of the fertile window may still result in conception, if timing is right and sperm can survive long enough), homosexual sex can obviously never result in conception, making its prevalence particularly interesting with respect to this manuscript. While an in-depth review of all the current research into the potential adaptive functions of human same-sex sexual behaviour are outside the scope of this work, we find it interesting that in humans, homosexual behaviour has been proposed to have adaptive social functions via a socio-sexual hypothesis [95], which we can also apply here to non-human primates. In particular, when we consider the above potential function of non-conceptive sex as a form of communication, we can see how social reinforcement, alliance formation, and conflict resolution (all offered as potential adaptive, prosocial benefits of homosexual behaviour: reviewed in [95]), may be said to function communicatively, and therefore provide evidence for a prosocial, communicative function of homosexuality in other primates too. If same-sex sexual behaviours were more prevalent in primate societies with higher perceived levels of prosocial behaviour, such as chimpanzees, bonobos and capuchins [96,97], or cooperative breeders, such as marmosets and tamarins (reviewed in [97]), as opposed to those expected or perceived to have lower levels of prosocial behaviour, for example solitary primates like galagos and orangutans, this could begin to support such a theory. Evidence already exists that bonobos exhibit more same-sex sexual behaviour in specific contexts than chimpanzees do in the same contexts (reviewed in [95]), and we know that neurologically, bonobos appear to be wired for more prosocial behaviours than chimpanzees [98], suggesting that homosexuality and prosociality could occur along a continuum.

It is clear that more research is needed to tease these potential influences apart. Potential studies could focus on comparing mating and social systems across primate taxa and cross-referencing these with reported instances of non-conceptive sex (including separate analyses for homosexual sex, where appropriate), with information on context, as well as rank and relationship between individuals. These data may help determine whether non-conceptive sex is more common among certain primate groups and whether potential explanations for sex, detailed in this commentary, are dependent on the systems themselves. Future primary data collection of this type will be needed to confirm primate societies where non-conceptive sex has not been reported, or has been under-reported. In addition, patriarchal and matriarchal societal influences require careful consideration, as these likely interplay with multiple other factors, as described above. That being said, mining the considerable existing literature on primate sociality may provide useful information in this regard, as well as a clearer direction for future investigations.

## 7. Conclusions

Recent evidence that non-human primate sex occurs for pleasure, communication, dominance, and avoidance of infanticide, as well as to produce offspring, challenges the conventional view that reproductive sex is the only major player in determining the sexual behaviours of individual primates, and is therefore at the forefront of determining the eventual structure of primate societies. While sexual attraction and sexual behaviours undoubtedly drive these facets of primate social dynamics, they do so even without (sometimes explicitly without) the promise of resultant offspring. In humans, sexuality is driven by much more than reproduction, yet if the same is also true for primates (and other non-human animals), as we argue here, that strips down yet another of the barriers between us and them. 

We conclude that there is a need for more research on non-conceptive sex in primates, and that alongside the evidence presented here, we predict that primates, and probably other sexually active animals, engage in sex for multiple reasons, of which reproduction is just (a subconscious) one.

The ubiquity of non-conceptive sex among primates suggests that pleasure, dominance, communication, and probably other unexplored functions of sex have been co-opted to make sexual behaviour worth the costs and unpredictability of its reproductive outcome. We propose that primate sex, like human sex, should be examined with an eye towards these other non-conceptive functions. By shedding the constraints of the traditional “sex-for-reproduction-only” model, the richness of primate behaviour can be made more accessible and open to new interpretation. We suggest that these views should be made available in assigned texts and classes to students of primate behaviour in higher education settings and that they also may provide future inspiration for scholars of animal behaviour. Ethology as a whole is perhaps not immune to the prejudices of past human societal and cultural norms, based on outdated perceptions of culturally appropriate sexuality and sexual behaviours. A more nuanced understanding and acceptance of the potential roles of non-conceptive sex among non-human primates is, therefore, overdue and its future study should be informed by the sexual complexities seen in modern human societies.

## Data Availability

Not applicable.
